# Poly C Binding Protein 1 Regulates p62/SQSTM1 mRNA Stability and Autophagic Degradation to Repress Tumor Progression

**DOI:** 10.3389/fgene.2020.00930

**Published:** 2020-08-14

**Authors:** Wenliang Zhang, Shaoyang Zhang, Wen Guan, Zhicong Huang, Jianqiu Kong, Chunlong Huang, Haihe Wang, Shulan Yang

**Affiliations:** ^1^Translational Medicine Centre, The First Affiliated Hospital, Sun Yat-sen University, Guangzhou, China; ^2^Department of Biochemistry, Zhongshan School of Medicine, Sun Yat-sen University, Guangzhou, China; ^3^Department of Hepatobiliary Surgery, The First Affiliated Hospital, Sun Yat-sen University, Guangzhou, China; ^4^Center for Stem Cell Biology and Tissue Engineering, Sun Yat-sen University, Guangzhou, China; ^5^Guangdong Engineering and Technology Research Center for Disease-Model Animals, Sun Yat-sen University, Guangzhou, China

**Keywords:** PCBP1, p62/SQSTM1, autophagy, apoptosis, ovary cancer, colon cancer, caspase-8

## Abstract

Accumulating evidence show that Poly C Binding Protein 1 (PCBP1) is deleted in distinct types of tumors as a novel tumor suppressor, but its tumor suppression mechanism remains elusive. Here, we firstly describe that downregulation of PCBP1 is significantly associated with clinical ovarian tumor progression. Mechanistically, PCBP1 overexpression affects various autophagy-related genes expression at various expression levels to attenuate the intrinsic cell autophagy, including the autophagy-initiating ULK, ATG12, ATG7 as well as the bona fide marker of autophagosome, LC3B. Accordingly, knockdown of the endogenous PCBP1 in turn enhances autophagy and less cell death. Meanwhile, PCBP1 upregulates p62/SQSTM1 via inhibition p62/SQSTM1 autophagolysome and proteasome degradation as well as its mRNA stability, consequently accompanying with the caspase 3 or 8 activation for tumor cell apoptosis. Importantly, clinical ovary cancer sample analysis consistently validates the relevance of PCBP1 expression to both p62/SQSTM1 and caspase-8 to overall survival, and indicates PCBP1 may be a master player to repress tumor initiation. Taken together, our results uncover the tumorigenic mechanism of PCBP1 depletion and suggest that inhibition of tumor cell autophagy with autophagic inhibitors could be an effective therapeutical strategy for PCBP1-deficient tumor.

## Introduction

Poly C binding protein 1 (PCBP1) as an RNA binding protein is widely involved in different gene regulatory levels, which include gene transcription, translation, RNA transportation splicing and posttranscription (Giles et al., 2003; Nishinakamura et al., 2007; Ravikumar et al., 2010; Wang et al., 2010, 2018, 2019; Tripathi et al., 2016; Zhang et al., 2017a, b; Ishii et al., 2018; Shi et al., 2018; Zhao et al., 2019). Recently, PCBP1 as a novel tumor suppressor is characterized to be downregulated in many cancer types on inhibition of tumor formation and metastasis (Guo and Jia, 2018), including gastric cancer (Ji et al., 2017) and thyroid cancer (Ji et al., 2017). We have uncovered that PCBP1 delays the translation of metastatic PRL-3 which is broadly downregulated in variety of tumors, indicating that PCBP1 could be a potential tumor suppressor (Wang et al., 2010). In 2017, Jiani Guo et al. (2017) reported that PCBP1 mediates drug resistance in colorectal cancer. It was reported that PRL-3 enhances autophagy and promotes cell proliferation under nutrient-efficient and nutrient-poor conditions (Huang et al., 2014). Following this information, we then disclosed that PCBP1, as the suppressor of PRL-3, can really inhibit the starvation-induced autophagy of tumor cells to block tumorigenic initiation, independent of PRL-3 (Zhang et al., 2016), but we still do not know whether PCBP1 participates in the normal basal autophagy process in the nutrition-efficient situation. We recently showed that PCBP1 also increases cell cycle inhibitor, p27^Kip1^ expression via its RNA binding capability to repress tumor cell cycle progression (Shi et al., 2018). Together with the previously mentioned, PCBP1 seems to work on multiple facets to inhibit tumor initiation and progression, but the underlying detailed mechanism remains elusive.

Autophagy is an evolutionally conserved process, in which the double-membrane vesicles (autophagosomes) initiated from multiple autophagy-related gene (ATG) products swallow and digest the damaged cytoplasmic organelles or proteins through lysosome-dependent degradation. Autophagy is putatively known as a cytoprotective response to cell stress for cell survival from cell death under stresses (Degenhardt et al., 2006; Villa et al., 2017). In contrast, this process could contribute to cell demise (autophagic cell death) when last too long time or works on overdose (Mizushima, 2007; Maiuri et al., 2010; Bialik et al., 2018). Many stresses can induce autophagy, which are composed of starvation, hypoxia and rapamycin inductions (Klionsky et al., 2012). Microtubule-associated protein Light Chain 3B (LC3B) is well known as the mammalian homolog of yeast *Atg8.* During autophagy, the LC3B protein undergoes modification from LC3B I to LC3B II served as a hallmark of formation (Klionsky et al., 2012). The p62/SQSTM1 (p62) protein is a link formed between LC3B II and autophagic substrates. p62 usually incorporates into the integrated autophagosome and can be subsequently degraded in autolysosomes, when autophagy process fully accomplishes (Bjorkoy et al., 2005; Pankiv et al., 2007). Thus, initiative autophagic flux can be indicated by LC3B-II amount and the accomplishment by p62 degradation status, respectively (Klionsky et al., 2012). The current results indicated that autophagy has dual roles in either promoting tumor initiation or inhibiting tumor progression (Levine, 2007; Mizushima, 2007; Galluzzi et al., 2015; Singh et al., 2018). Generally, tumor cells in tumor mass center are lack of nutrition, thus have higher autophagic flux than those in tumor margin regions, to prevent their death (Degenhardt et al., 2006). From another way, autophagy also exists in dying cells to result in the eventual cell death through the excessive consumption of cellular components (Janku et al., 2011; Young et al., 2012; Huang et al., 2013). Thus, roles of autophagy in tumorigenesis are highly dependent on pathological and physiological conditions of cell context and microenvironment. So far, it remains elusive whether PCBP1 modulates and participates in tumor cell autophagy in the nutrition-efficient condition.

Apoptosis is a form of programmed cell death and characterized by the cascade activation of caspases (Fulda and Debatin, 2006; Li and Yuan, 2008; D’Arcy, 2019). Caspase-8 is an initiator caspase in apoptosis. The auto-activation of caspase-8 starts from its oligomerization and self-cleavage. Subsequently, the activated caspase-8 facilitates the activation of pro-caspase-3, which is an executioner caspase, and promotes the apoptotic cleavage of poly (ADP-ribose) polymerase (PARP) for apoptosis (Kruidering and Evan, 2000). Recent reports suggested that cross talking between autophagy and apoptosis can coordinately regulate cell fate (Wu et al., 2014). As mentioned above, PCBP1 can suppress tumorigenesis, but we still also do not understand if it is related to tumor cell death.

Our results suggest that PCBP1 not only downregulates autophagic flux in the starvation conditions by suppressing LC3B expression as previously reported (Zhang et al., 2016), but also coordinately represses multiple autophagic genes expression, including ULK1, ATG7, ATG12 and p62 to suppress tumor cell autophagy initiation and commitment, and eventually to enhance tumor cell apoptosis. Thus, the expression states of PCBP1, p62 and Caspase-8 could be predictive biomarkers, and the anti-autophagic approaches would be potential therapeutical strategy for patients with silence of PCBP1 and high autophagic flux.

## Materials and Methods

### Cell Culture and Treatment. Human Ovarian Cancer Cell Lines

SK-OV3 and A2780, Chinese hamster ovarian cell line (CHO), human colorectal cancer cell lines DLD-1 and HCT-116 were purchased from the ATCC company and maintained as previously reported by our group (Shi et al., 2018). Unless otherwise specified, chloroquine diphosphate salts (CQ, 50 μM 24 h, Sigma-Aldrich), 3-Methyladenine (3-MA, 3 μM, 12 h, Selleck, China), actinomycin D (Act D, 0.5 μg/ml, Biosharp, 8 h or 12 h) and MG132 (20 μM, 12 h, Sigma-Aldrich) was, respectively, dissolved in PBS or DMSO, and used to inhibit autophagic degradation, terminate the novel transcription or inhibit protein degradation.

### DNA Constructs and Transfection

The A2780-PCBP1, DLD-1-PCBP1, HCT-116-PCBP1, and SK-OV3-PCBP1 stable cell lines were established as previously described (Wang et al., 2010). Four specific shRNA constructs as previously described (Zhang et al., 2016) for knockdown of PCBP1. The cell transfections were performed out with the Lipofectamine 2000 reagent (Invitrogen) as the manufacturer’s instructions.

### Antibodies and Western Blots (WB)

Antibodies were used against the following: PCBP1 (Cat No. sc-137249, Santa Cruz, WB 1:500); p62/SQSTM 1 (Cat No. 5114, Cell Signaling Technology (CST), WB 1:1000); LC3B (Cat No. 3868, CST, WB 1:1000, IF 1:200); Caspase-8 (Cat No.13423-1-AP, Protein Tech, China, WB 1:500); Caspase-3 (Cat No. 9662, CST, WB 1:1000); PARP (Cat No. 9542, CST, WB 1:1000); c-caspase-3 (Cat No. 9661, CST, WB 1:1000); ULK1 (Cat No.4773, CST, WB 1:1000); c-PARP (Cat No. 9541, CST, WB 1:1000); ATG7 (Cat No.8558, CST, WB 1:2000); ATG12 (Cat No.4180, CST, WB 1:1000); ATG5 (Cat No.2630S, CST, WB 1:1000); β-Actin (Cat No.4967, CST, WB1:2000); anti-mouse HRP-labeled secondary antibody (Cat No. 7076S, CST, WB 1:2000), GAPDH (Cat No. CW0101A, CW Bio-tech, China, WB 1:2000) and anti-mouse HRP-labeled secondary antibody (Cat No. 7076S, CST, WB 1:2000). The western blots protocols were followed as previously reported by our group (Zhang et al., 2016). Protein band intensity was, respectively, quantified and analyzed with densitometry by using Image J software.

### Calculating Densitometry of Immunoblots

The quantitative densitometry of immunoblots was carried out as previously described (Zhang et al., 2016). Protein band intensity was respectively quantified and analyzed by using Image J software. Relative protein levels were calculated using densitometry values for GAPDH or β-actin as calibrators and shown under the protein bands.

### Immunofluorescence Microscopy

For immunofluorescence, cells were seeded and grown on cover slips. And then, cells were washed in 1 X PBS and fixed in 4% paraformaldehyde (PFA). After permeabilization with 0.2% Triton X-100 (Biosharp), cells were probed with LC3B primary antibodies (CST, Cat No. 3868, IF 1:200) followed by anti-rabbit Alexa Fluor 594. Subsequently, cells were incubated with DAPI and mounted. Cells were observed by LSM710 confocal microscopy (Carl Zeiss AG).

### Autophagy Assays

Autophagic flux was detected based on the amount of endogenous LC3B puncta, and validated with p62 protein level for autophagy completion. Cells were pre-treated with 50 μM CQ and DMSO for indicated time, respectively. The autophagy assay protocols are followed as previously reported by our group (Zhang et al., 2016).

### Reverse Transcriptase-Polymerase Chain Reaction (RT-PCR)

5 μg of RNA was reverse transcribed to cDNA by using M-MLV Reverse Transcriptase (Promega, Cat No. M1705) following the manufacturer’s instruction. Gene-specific primers used for RT-PCR amplification of p62, ULK1, ATG 12, ATG 7, PRL-3 and internal control GAPDH were listed in Supplementarys Table 1. The RT-PCR amplification is performed by using GoTaq^®^ DNA polymerase (Promega, Cat No. M3005) in the amplification system, according to the manufacturer’s protocol. DNA band intensity was quantified with densitometry via Image J software.

### Flow Cytometry Apoptosis Analysis

To investigate the impact of PCBP1 overexpression on the tumor cell apoptosis, A2780-PCBP1 and A2780-GFP cells were seeded and cultured overnight, followed with treatment of 3 μM Paclitaxel or DMSO for 27 h, respectively. Next, adherent cells were trypsinized with 0.25% EDTA-free trypsin and stained with an APC-conjugated Annexin V and 7-ADD kit (KeyGEN BioTECH, China) following the manufacturer’s instructions. The percentage of apoptotic cells were quantified by flow cytometer (Gallios, Beckman, United States).

### mRNA Stability Assay

A2780-PCBP1 or GFP control cells were treated with DMSO and 0.5 μg/ml Act D (Biosharp) for 8 and 12 h, respectively. Five microgram of RNA was used for cDNA synthesis, and the transcript abundances of p62 mRNAs and GAPDH mRNA controls were quantified via semi-quantitative RT-PCR. The primers of p62 and GAPDH for RT-PCR was provided (Supplementary Table 1).

### Ribosome Profiling of Autophagy Genes

Ribosome profiling protocols were followed as previously reported by our group (Shi et al., 2018). ULK1, ATG12, ATG7, PRL3, GAPDH mRNA was detected by qRT-PCR with the primers (Supplementary Table 1) as above.

### Cell Proliferation Assay

Cells were seeded in 96-well plates in triplicates and cultured overnight. Then, cell culture medium was replaced with 100 μl serum-containing media with or without CQ treatment at the indicated time points. After treatments, cell counting kit-8 (CCK8) reagent (BB-4202-1, Best Bio, China) was added in each well (10 μl per well) and the cells were incubated for another 2 h. The absorbance at 450 nm of each well was measured by enzyme-labeling instrument (Multiskan G0; Thermo Fisher Scientific, Inc.).

### Immunochemistry (IHC) Analyses of PCBP1, p62, and Caspase 8 Expressions in Ovary Tumor Samples

Human freshly frozen colon tumor tissues were collected from Sun Yat-sen University Cancer Center, under their Standard Experimental Ethics Protocol and approved by Sun Yat-sen University Research Ethics Committee. Three ovary tissue arrays were purchased from Xi ’an Ailina Biotechnology Co., Ltd., under their Standard Experimental Ethics Protocol. These tissue arrays were stained with anti-PCBP1 (1:200 dilution, Abcam, United States), anti-p62 (1:200 dilution, Sanying Biotechnology Co., Ltd., Wuhan, China) and anti-Caspase-8 (1:200 dilution, Protein Tech, China) antibodies, respectively. Immunochemistry protocol and the semi-quantitative score was followed as previously reported by our group (Shi et al., 2018). The semi-quantitative score is presented as Score = SI (staining intensity) x PP (percentage of positive cells), in which SI was determined as ten levels including 0 (negative); 1; 2; 3; 4; 5 (Supplementary Figure 1). Likewise, PP was defined as 0, < 5%; 0.2, 6%–30%; 0.5, 31%–70%; and 1.0, > 70% positive cells. As an example, the relative score in Supplementary Figure 1 based on IRS are determined as 0, 1, 2, 3, 4, and 5.

### Statistical Analysis

Statistical analyses were performed by GraphPad Prism software (Version 5.00). Unless otherwise specified, the unpaired *t-*tests and Analysis of Variance (ANOVA) analysis were used to compare two groups and multiple groups, respectively. *P* < 0.05 were considered as significant. ^∗^*P* < 0.05; ^∗∗^*P* < 0.01; ^∗∗∗^*P* < 0.001.

## Results

### PCBP1 Is Downregulated and Negatively Related to Tumor Malignancy in Ovarian Tumors

**FIGURE 1:**
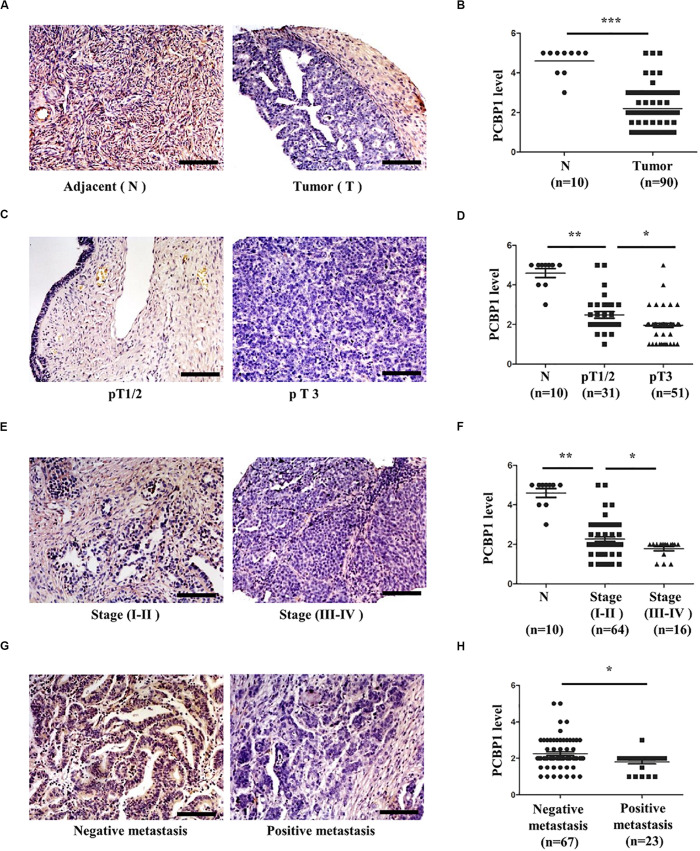
PCBP1 expression is negatively related to ovary cancer progression. **(A)** Representative immunohistochemistry (IHC) analysis of PCBP1 expression in the paired adjacent normal tissues (N) and their ovarian tumor samples (T). **(B)** Statistical analysis of PCBP1 expression between normal and tumor tissues detected in **(A)**. **(C)** Representative PCBP1 expression level in pT1/2 tumors (with or without pelvic spread) vs. pT3 tumor (with micro-metastasis of extra pelvic peritoneum confirmed by microscope). **(D)** Statistical comparison of PCBP1 expression between the pT1/2 tumors and the pT3 tumors detected in **(C)**. **(E)** Representative PCBP1 expression in tumor samples with different tumor progression (clinical stages). **(F)** Statistical analysis of PCBP1 expression with tumor progression (clinical stages). **(G)** Representative PCBP1 expression in primary tumor samples and the metastatic tumor samples. **(H)** Statistical analysis of PCBP1 expression between the primary and metastatic ovary cancer samples. All IHC images are photographed with 200X amplification. Scale bars equal to 100 μm. *P < 0.05; **P < 0.01; ***P < 0.001.

We previously reported that PCBP1 is broadly downregulated in lung and colon cancers (Wang et al., 2010). PCBP1 downregulation promotes cell proliferation and tumorigenicity of ovarian cancer cells both *in vitro* and *in vivo* (Zhang et al., 2016; Shi et al., 2018). To further confirm the clinical relevance of PCBP1 to ovarian cancer progression, we examined PCBP1 expression state by immunohistochemistry (IHC) in 90 cases of ovarian cancer, compared with the 10 corresponding cancer adjacent ovarian tissues among them. Our IHC results were scored (Supplementary Figure 1) and indicated that PCBP1 was more detectable in cancer adjacent tissues than ovarian cancer samples (Figures 1A,B). Among ovarian cancer cases, according to tumor node metastasis (TNM) classification, PCBP1 levels were especially lower in pT3 group (tumor with micro-metastasis of extra pelvic peritoneum confirmed by microscope) vs. pT1/2 group (tumor with or without pelvic spread) (Figures 1C,D), as well as the late clinical stage (stage III and IV) vs. early clinical stages (stage I and II) based on clinicopathologic features (Figures 1E,F). Statistically significant decreased PCBP1 expression was also found in ovarian cancer samples with positive lymph node and distant metastasis statues, compared with those with negative status (Figures 1G,H). Moreover, we summarized the correlation between PCBP1 expression and clinicopathologic variables of patients with ovarian cancer (Table 1). In addition, our immunohistochemical staining results also suggested that PCBP1 protein expression is significantly decreased in tumor regions of colorectal cancer compared with the paired fresh normal tissues (Supplementary Figure 2; Shi et al., 2018). The overall analysis showed that low PCBP1 levels were significantly associated with tumor stages (*p* < 0.01), clinical stages (*p* < 0.05), lymph node and distant metastasis (*p* < 0.05).

**TABLE 1:** PCBP1 expression and clinicopathologic characteristics of ovarian cancer patients.

	PCBP1			
Characteristics	Low	Median	High	*p*-value
**Pathology**				
Adjacent	–	1	9	<0.0001
Tumor	70	13	7	
**Ages**				
= 46	23	5	5	0.6748
>46	47	9	11	
**Tumor stage**				
T _1__+__2_	19	8	4	0.0062
T _3__+__4_	44	5	2	
**Clinical stage**				
I + II	46	13	5	0.0317
III + IV	16	–	–	
**Metastasis**				
Negative	49	13	5	0.0177
Positive	22	1	–	

**t-test was used. PCBP1 low means IHC score as 1 and 2; Median, 3; High, 4 and 5. Refer to Supplementary Figure 1A.**

### PCBP1 Inhibits the Basal Intrinsic Autophagy in Tumor Cells

**FIGURE 2:**
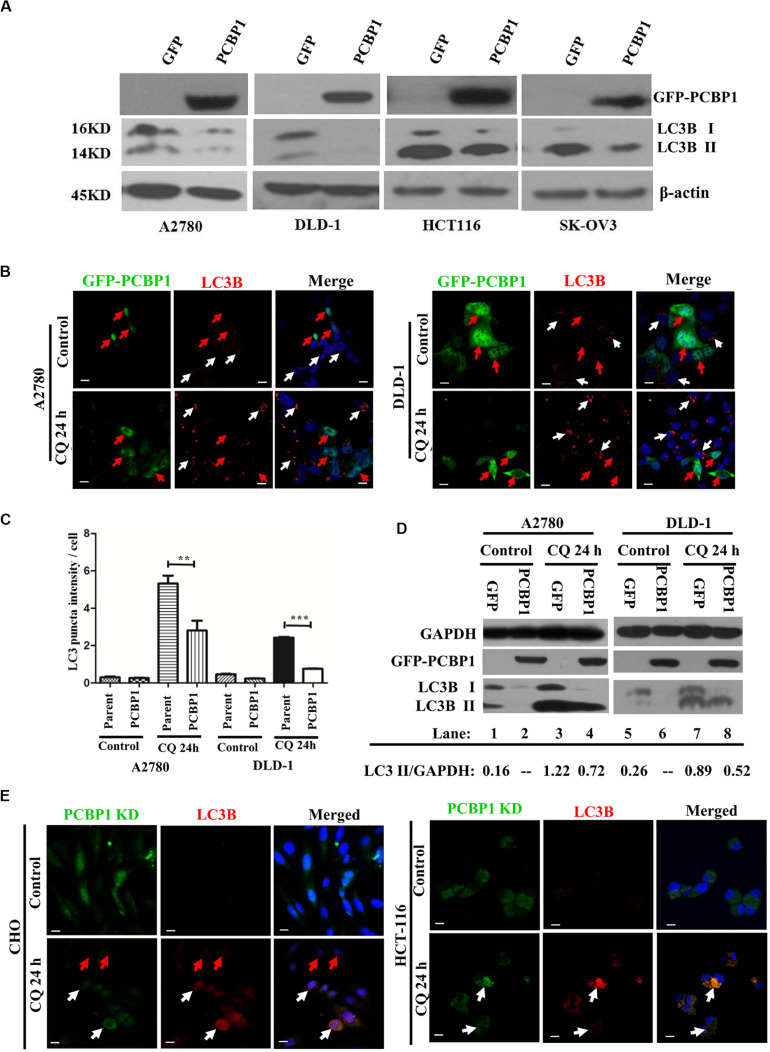
PCBP1 Overexpression represses autophagy. **(A)** Immunoblots of PCBP1, LC3B I and II in PCBP1-overexpression (GFP-PCBP1) and the GFP control cells. β-actin is used as an internal loading control. **(B)** Immunofluorescence staining of LC3B puncta in both A2780 and DLD-1 cells with or without CQ treatment for 24 h. GFP-PCBP1 cells are in green, while parent control cell are not green. Cells counterstained with DAPI are shown in blue. White arrows point LC3B puncta (Red) in the parental control cells without GFP-PCBP1 transfection (not green), while the red arrows point LC3B puncta in GFP-PCBP1-transfected cells (green). Bars equal to 25 μm. **(C)** Quantifications of LC3B puncta intensity per cell in B are presented as histograms (mean ± SD). Parent group indicates the parent control cells without GFP-PCBP1 (not green), while the PCBP1 group indicates the cells with transfected GFP-PCBP1 (green). **P < 0.01; ***P < 0.001, *n* = 5. **(D)** Immunoblots of LC3B I, II in A2780 and DLD-1 cells with PCBP1 and GFP overexpression. Upon CQ or DMSO (control) treatment for 24 h, cells were analyzed. Immunoblot intensity ratio of LC3B II to GAPDH were, respectively, quantified and normalized, and indicated in each lane. **(E)** Immunofluorescence staining of LC3B puncta in both A2780 and DLD-1 cells with or without CQ treatment for 24 h. PCBP1 knockdown (KD) cells are in green, while parent control cells are not green. Cells counterstained with DAPI are in blue. White arrows point LC3B puncta (Red) in the PCBP1 KD cells, while the red arrows point LC3B puncta in the parental control cells. Scale bars in **(B,E)** equal to 25 μm.

Although we have shown that PCBP1 inhibits autophagy to repress cell proliferation in nutrition-deficient condition via downregulation of LC3B, a key gene of autophagy degradation (Zhang et al., 2016), but it is still not well understood that whether PCBP1 is also involved in the intrinsic basal autophagy regulation to impair tumorigenesis. To thoroughly answer this question, we conducted immunoblotting and showed that, compared with the control cells transfected with empty GFP vector, GFP-PCBP1 overexpression an obviously decreased the expression of LC3B I and LC3B II in A2780, DLD-1, HCT-116, and SK-OV3 cells from different tissue origin in nutrient-rich condition (Figure 2A). These results also indicate the existence of intrinsic autophagy event in various tumor cells, which could be regulated by PCBP1 overexpression. To investigate the fundamental function of PCBP1 in the basal autophagy regulation, immunofluorescence staining is performed and further validated the reduced aggregated LC3B puncta by PCBP1 overexpression upon an autophagy degradation inhibitor, chloroquine (CQ) treatment in both A2780 and DLD-1 cells under nutrition-rich conditions (Figure 2B). The LC3B puncta intensity in a cell is quantified and results demonstrate that PCBP1 overexpression cells have dramatically less LC3B puncta intensity than the parent control cells (Figure 2C). In addition, we noted that the quantified results of LC3B puncta intensity in Figure 2C did not significant decrease in the PCBP1 overexpression group compared with the control group, but the immunoblotting results in Figure 2A showed a significant decrease in LC3B expression. We think that the possible reason is due to the sensitivity of LC3B antibody that is not so sensitive for immunofluorescence detection, in comparison to immunoblotting detection. In line to this, immunoblotting results indicated that PCBP1 overexpression downregulates both types of LC3B I and II expression in full-nutrition situation, compared with its GFP control cells (Figure 2D, lane 1 vs. 2 and lane 5 vs. 6). Whereas, upon CQ treatment, LC3B levels were not completely recovered to the control GFP level (Figure 2D, lane 3 vs. lane 4 and lane 7 vs. lane 8), which is consistent with our previous results that PCBP1 downregulates LC3B expression independently of autophagic degradation (Zhang et al., 2016).

To verify if endogenous PCBP1 really participate in basal autophagy regulation, endogenous PCBP1 was knocked down with 4 specific shRNAs in A2780, CHO, and HCT-116 cells with relatively high PCBP1 expression (Supplementary Figures 3A,B). Immunofluorescence staining revealed the increased aggregation of LC3B puncta intensity by deletion of endogenous PCBP1 upon CQ treatment in both CHO and HCT-116 cells under nutrition-efficient conditions (Figure 2E). CHO cells are relatively normal cells compared with the ovarian cancerous cells. Therefore, the results from CHO cells can evidently indicate the intrinsic autophagy event in normal ovary cells (tissues) to further show the general information. Likewise, deletion of PCBP1 robustly boosted the modified LC3B-II accumulation, which indicated as the ratio of LC3B-II to GAPDH in A2780 cell line (Supplementary Figure 3C, lane 1 vs. lane 2) in nutrition-rich conditions. After CQ treatment, LC3B levels were still more than the control level, demonstrating downregulation effect of LC3B by PCBP1 deletion (Supplementary Figure 3C, lane 3 vs. lane 4, lane 5 vs. lane 6). In this study, although the ovary cancer here is focused, the consistent results observed in CHO cell line and two colon cancer cell lines could to suggest the general observation of PCBP1 in autophagy modulation. Taken together, our results verified that PCBP1 downregulates LC3B expression to result in basal autophagy inhibition.

### PCBP1 Upregulates p62/SQSTM1 Through Inhibiting Its Autophagic Degradation

**FIGURE 3:**
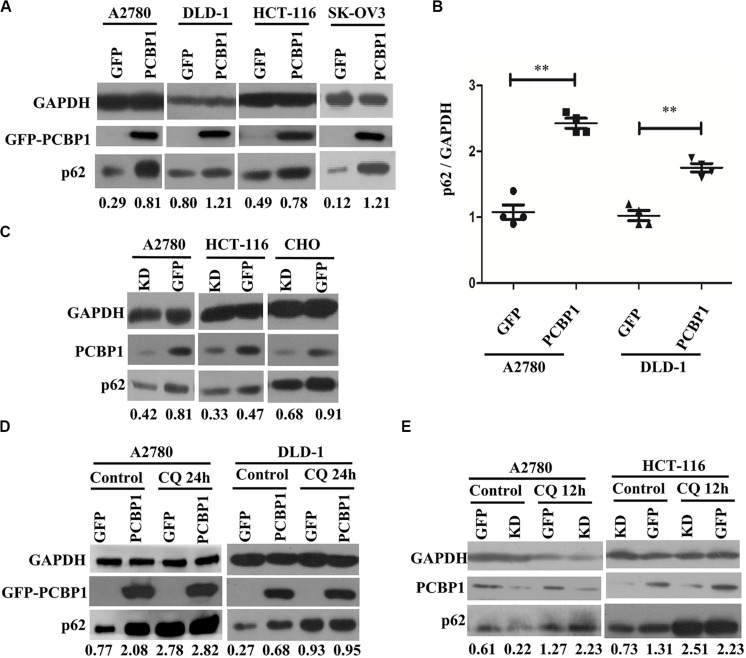
PCBP1 inhibits autophagy completion. **(A)** Immunoblots of p62 and GAPDH in the PCBP1 overexpression (GFP-PCBP1) and the GFP control cells by at least two independents. **(B)** Statistical analysis of p62 expression in PCBP1 overexpressing cells, compared with the GFP control cells by at least three independent immunoblots. ^∗∗^*p* < 0.01. **(C)** Western blots of p62 in the indicated endogenous PCBP1 knockdown cells. Four GFP-confused shRNAs against PCBP1 were transfected into cells to establish the PCBP1 knockdown cells, while the GFP empty vector-transfected cells are control cells. **(D)** Immunoblots of p62 in A2780 and DLD-1 cells with PCBP1 and GFP overexpression. Upon CQ or DMSO (control) treatment for 24 h, cells were analyzed by at least two independents. **(E)** Immunoblots of p62 in A2780 and HCT-116 cells with PCBP1 knock down (KD) by at least two independents. GAPDH is used as an internal loading control, and the expression ratio of p62 to GAPDH was quantified and normalized, and indicated under each lane.

Considering that LC3B expression can be suppressed by PCBP1 (Zhang et al., 2016), and it may not suitable to evaluate the influence of basal autophagic flux by PCBP1 with LC3B accumulation. In addition, autophagy is a complex, dynamic process, and LC3B-II accumulation just reflects the autophagic initiation, not the performance outcome (Ravikumar et al., 2010). Thus, p62/SQSTM1 (p62) was used to examine PCBP1’s influence in the basal autophagic flux, as p62 is the well-known substrate of autophagic degradation. According to this concept, we determined p62 level and western blotting results presented that, compared with the GFP control cells, p62 expression was robustly increased in various types of PCBP1-overexpressing cells under normal condition (Figure 3A). Quantification of p62 also confirmed a significantly higher p62 expression in PCBP1 overexpressing A2780 and DLD-1 cells compared with their GFP control cells, respectively (Figure 3B). On the contrary, endogenous PCBP1 depletion evidently decreased p62 protein level in A2780 cell line, HCT-116 cell line and CHO cell lines (Figure 3C). When inhibiting the autophagy completion with CQ, p62 level could be almost restored back to that of the parental control cells in DLD-1 cells, but not in A2780 (Figure 3D) exceptionally. Accordingly, CQ treatment similarly reversed p62 level in the PCBP1 depletion cells back to that in normal GFP control level (Figure 3E), suggestive of the p62 regulation by PCBP1 is at least an autophagy-dependent manner.

### p62 Is Also Upregulated via Proteasome Degradation Inhibition and mRNA Stabilization by PCBP1

**FIGURE 4:**
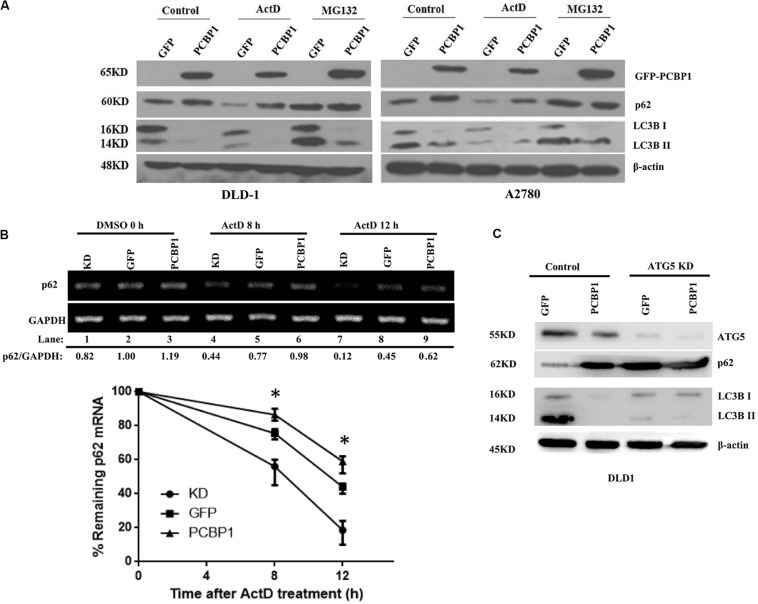
PCBP1 enhance p62 expression in multiple levels. **(A)** Immunoblots of p62 and LC3B in the indicated GFP-PCBP1 cells as well as their parental cells treated without (Control) or with Actinomycin D (ActD) or MG132, respectively. **(B)** Semi-quantitative RT-PCR analysis of p62 mRNA stability (upper panel) in A2780 cells with endogenous PCBP1 knockdown (KD) or PCBP1 overexpression (PCBP1), compared with control cells (GFP) by at three independents. GAPDH is used as an internal control, and the ratio of p62 mRNA level to GAPDH was quantified, normalized and indicated under each lane. *P < 0.05. **(C)** Immunoblot of p62 protein level in DLD-1 cells with or without ATG5 knockdown by at least two independents. GAPDH was used as an internal loading control.

Comparing p62 protein level between the CQ inhibited parental control cells and PCBP1-overexpressing cells, it seems that p62 could be modulated not only by the autophagy-dependent degradation, since CQ treatment did not restore p62 expression back to the normal level. We further investigated which other mechanism may be involved in p62 regulation. We treated cells with Act D or MG132 to block the novel mRNA transcription or protein proteasome degradation, respectively. Immunoblots showed that, after MG132 (a known proteasome inhibitor) treatment, p62 expression was obviously increased in the GFP cells, but not in GFP-PCBP1 overexpressing cells, compared with the corresponding control groups (Figure 4A), indicating PCBP1 can somehow inhibit proteasome-mediated p62 degradation. As PCBP1 is an RNA-binding protein as we have shown, we further carried out a semi-quantitative RT-PCR detection of p62 mRNA level, and our results indicated that PCBP1 overexpression led to more p62 mRNA amount, whereas endogenous PCBP1 knock down resulted in less p62 mRNA, compared with control cells (Figure 4B). To further clarify whether p62 mRNA amount alteration is due to its mRNA stability or transcriptional activation, we first blocked the novel mRNA synthesis with Act D treatment and detected stability of the newly synthesized p62 mRNA and its protein expression. Results showed that compared with that of control cells, PCBP1 delayed the degradation efficiency of the nascent p62 mRNA (Figure 4B). In addition, we knocked down the ATG5 expression in both DLD-1 GFP and PCBP1-overexpressing cells to reduce autophagic degradation and demonstrated that p62 protein was still high in the PCBP1-overexpressing cells, presenting the similar ratio to p62 mRNA level (Figure 4C). Altogether, the above results showed the authentic cause of p62 upregulation by PCBP1 is resulted from the multiple events, including the inhibitions of p62 autophagic and proteasome-mediated degradations, as well as the stabilized p62 mRNA level.

### PCBP1 Coordinately Regulates Multiple Autophagy Genes on Translational Level

**FIGURE 5:**
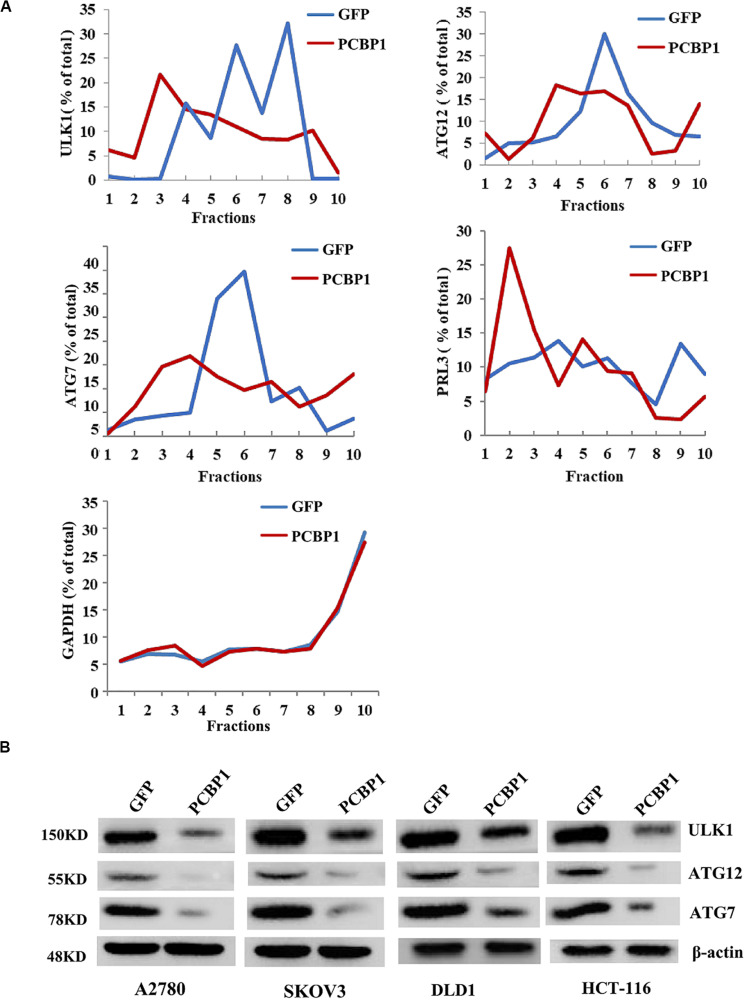
PCBP1 translationally enhance autophagy genes expression. **(A)** Polyribosome profiling of the indicated autophagy-promoting genes in A2780 cells with GFP-PCBP1 overexpression as well as the control GFP cells. As PRL-3 is translationally repressed by PCBP1 (Wang et al., 2010), it is used as positive control, GAPDH as a negative control. **(B)** Immunoblots of the indicated autophagy-promoting genes in various cell types with GFP-PCBP1 expression, compared with their GFP cells by at least two independents.

Given PCBP1 as an RNA-binding protein, it is involved in various levels of gene expression regulations, including transcription, splicing, translation and RNA stability (Giles et al., 2003; Wang et al., 2010; Zhang et al., 2010; Shi et al., 2018). To determine whether PCBP1 can regulate the autophagy-related genes at different levels, such as at the translational level, we conducted the gene expression analysis by PCBP1 overexpression as well as the ribosome profiling analyses of these genes’ translational status. Ribosome profiling results showed that PCBP1 clearly repressed ULK, ATG12, ATG7 translational efficiency, compared the known PCBP1 translationally repressed metastatic phosphatase PRL-3 (Wang et al., 2010), whereas the housekeeping gene GAPDH as an internal control was not affected (Figure 5A). In addition, the ATG12 and ATG7 were observed in our PCBP1-bound mRNA pool by immunoprecipitation (Supplementary Figure 4). To confirm the results, we did immunoblots and showed that these autophagy-related protein levels were clearly decreased accordingly (Figure 5B), validating the translational influence of PCBP1 in autophagy-initiating genes expression. Taken together, all above results suggested that PCBP1 represses autophagy through multiples target genes at various regulation levels.

### PCBP1 Coordinately Favors Tumor Cell Apoptosis

**FIGURE 6:**
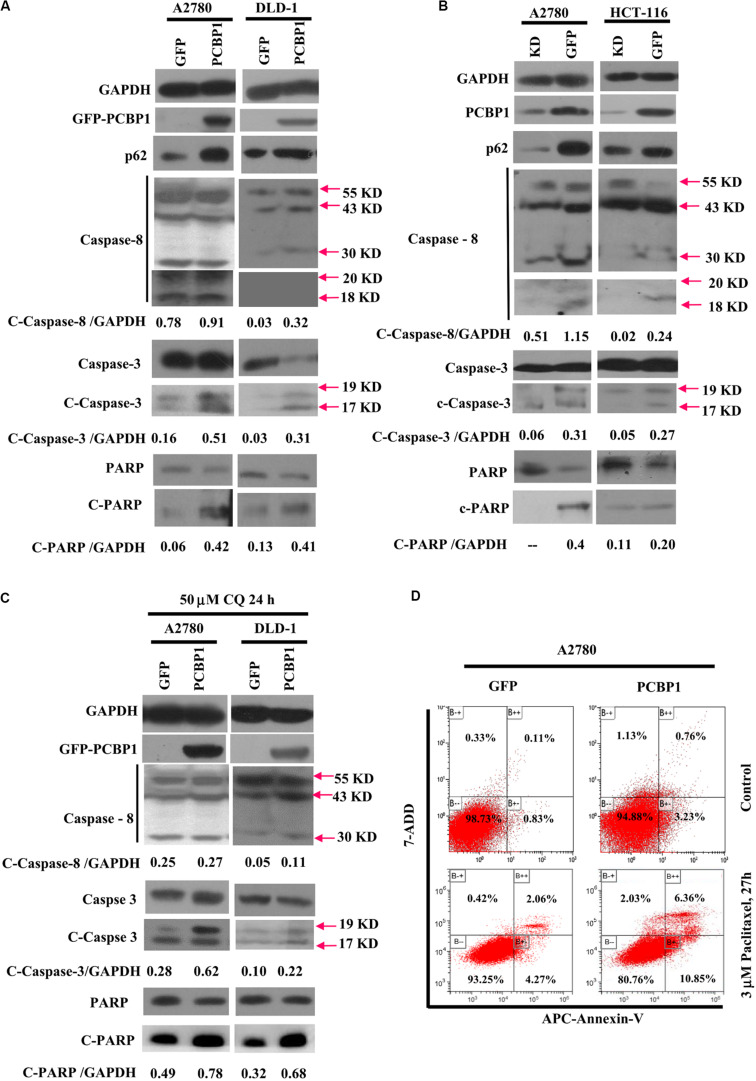
PCBP1 overexpression enhances caspases activation for apoptosis. **(A)** Immunoblots of p62, PARP and caspases and their cleaved forms in A2780 and DLD-1 cells with PCBP1 and GFP overexpression at least two independents. **(B)** Immunoblots of p62, PARP and caspases and their cleaved forms in A2780 and HCT-116 cells with endogenous PCBP1 knockdown (KD), compared with A2780 and HCT-116 GFP controls by at least two independents. **(C)** Upon CQ treatments for 24 h, immunoblots of PARP, caspases and their cleaved forms in A2780 and DLD-1 cells with PCBP1 and GFP overexpression at least two independents. Arrows point and show the molecular weight of each protein. The cleaved forms of these proteins are shown as c-protein. **(D)** Flow cytometric analyses of the percentages of apoptotic cells in A2780 cells with PCBP1 overexpression, compared with the A2780 GFP control cells by at least two independents. Overall percentages of apoptotic cells are defined as the sum of Annexin-V positive cells.

Given that autophagy plays different roles in tumorigenic outcome, to examine tumor cell fate upon autophagy retardation by PCBP1 in the normal culture conditions, we analyzed the apoptotic status of cells with PCBP1 aberrant overexpression. In line with our previous observation (Zhang et al., 2016), compared with the parent control cells, the PCBP1 aberrant expression obviously increased the cleaved-caspase-8 (c-caspase-8), cleaved-caspase-3 (c-caspase-3), and cleaved-PARP (c-PARP) levels in DLD-1 cells, as well as in A2780 cells with the exception of the caspase-8, which may be due to the cell type context specificity. Those results consistently suggested that PCBP1 could instinctively drive cell apoptosis, while suppressing autophagy (Figure 6A). On the contrary, the instinct apoptotic signals attenuated in both PCBP1 depletion cells (Figure 6B). Upon autophagy blockade with CQ treatment in nutrient-sufficient condition, the apoptotic status was increased at the relative level in the parental cells which is similar to that in PCBP1-overexpressing cells (Figure 6C), demonstrating that the high level of p62 induced by PCBP1 can somehow enhance apoptosis. This observation is in line with the notion that p62 is indeed involved in cell death regulation (Zhang et al., 2016; Tao et al., 2020). To confirm the physiological outcome, we conducted cell proliferation assay and showed that PCBP1 overexpression delayed cell proliferation in normal condition, compared with the control cells; while inhibition of intrinsic autophagy with CQ treatment in both the parental control cell and PCBP1 overexpressing cells efficiently repressed the cell proliferation, further indicating this intrinsic autophagy benefits to cell proliferation (Supplementary Figure 5). Apoptosis analysis also showed overexpression of PCBP1 could even favor tumor cell apoptosis in normal culture condition (Figure 6D). Together, our results clearly demonstrated that PCBP1 coordinately inhibits intrinsic tumor cell autophagy and favors cell apoptosis for tumorigenesis inhibition.

### Clinicopathological Correlation of PCBP1, p62, and Caspase-8 in Ovarian Cancer

**FIGURE 7:**
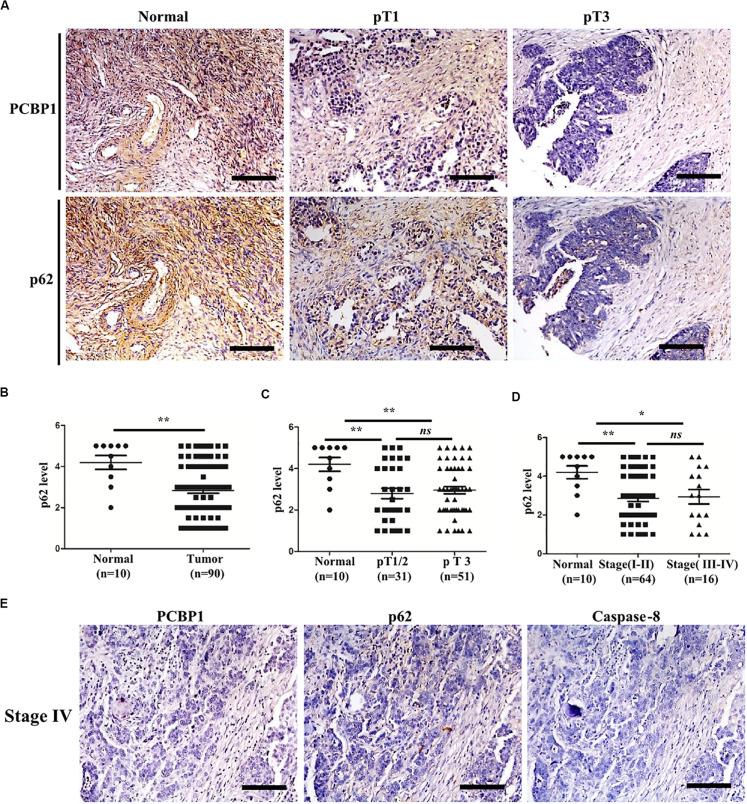
Relevance of PCBP1 to p62 and caspase 8 in ovary cancers. **(A)** Representative immunohistochemistry (IHC) analysis of p62 expression in the paired adjacent normal tissues and their ovarian tumor samples. pT1, tumor without pelvic spread; pT3, tumor with micro-metastasis of extra pelvic peritoneum confirmed by microscope. **(B)** Statistical comparison of p62 expression between normal and tumor tissues detected in **(A)**. **(C)** Statistical comparison of p62 expression between the pT1/2 group (tumors with or without pelvic spread) and the pT3 group (tumors with micro-metastasis of extra pelvic peritoneum confirmed by microscope). **(D)** Statistical analysis of p62 expression with tumor progression (clinical stage). **(E)** Representative PCBP1, p62 and caspase 8 expressions in a same ovary tumor sample with clinical stage IV. All images in are photographed with 200X amplification. Scale bars on equal to 100 μm. *P < 0.05; **P < 0.01; ns, no significance.

To validate if there is a general clinicopathological correlation between PCBP1 and p62, p62 expression was examined with IHC in another set of the serial sections of ovarian tissues. IHC results revealed that p62 was similarly detectable in ovarian cancer adjacent tissues, and subdued in ovarian tumor samples (Figure 7A and Supplementary Figure 6A). Moreover, similar to PCBP1 expression pattern in ovarian sample, we observed statistically significant p62 expression decrease in ovarian cancer samples vs. the adjacent samples (Figure 7B). However, there was no difference between pT3 group (tumor with micro-metastasis of extra pelvic peritoneum confirmed by microscope) vs. pT1/2 group (tumor with or without pelvic spread) tumors (*p* = 0.5920) (Figure 7C), late clinical stage (stages III and IV) vs. early clinical stages (stages I and II) (*p* = 0.8129) based on clinical features (Figure 7D), positive lymph node and distant metastasis statues vs. those with negative status (*p* = 0.9531) (Supplementary Figure 6B). Furthermore, we also observed that p62 is consistently higher in PCBP1 positive-adjacent tissue, while consistently lower in pT1 (without pelvic spread) and pT3 tumor tissue with downtrend (Figure 7A). To thoroughly validate the relationship between PCBP1, p62 and apoptosis in tumor samples, we use the same batch of ovary cancer samples to check these 3 protein expressions at same time (Figure 7E and Supplementary Figure 6C). IHC results also showed that PCBP1 expression was positively correlated with p62 expression (Supplementary Figure 6D, *R*^2^ = 0.506) and Caspase-8 expression levels (Supplementary Figure 6E, *R*^2^ = 0.447) in ovarian tumor samples, respectively. In line with the above observation, the correlation analysis indicated that p62 is positively correlated with Caspase-8 as well (Supplementary Figure 6F, *R*^2^ = 0.598), which is upregulated in ovarian tumor adjacent tissues and is downregulated in ovarian tumor tissues. Overall, we found that PCBP1, p62, and Caspase-8 expression levels were correlated with each other in ovarian cancers (Figure 7 and Supplementary Figure 6).

## Discussion

Accumulating evidence indicates that autophagy has complicated effects in tumorigenesis, which are dependent on the tumor developmental stage. For instance, it is documented that autophagy induction is well correlated to the worse prognosis in ovarian carcinoma (Zhao et al., 2014), but other study in turn indicates that autophagy accompanies higher overall survival of the ovary cancer patient, as high autophagic flux could sensitize the tumor cells to chemotherapy (Valente et al., 2014). Considering our previous result (Zhang et al., 2016), we assume that that once cells get transformed, the intrinsic autophagy would promote tumor cell survival in both nutrition-efficient and -deprived conditions, as long as the autophagy level (or intensity) is not too high. PCBP1 may naturally suppress or balance the intrinsic basal cell autophagy for cell homeostasis maintenance, as PCBP1 can affect various autophagy-related genes expression at the different autophagic stages, including the autophagy initiation-related genes ULK, ATG12 and ATG7, the autophagosome formation, LC3B, the autophagosome-lysome fusion, p62 in nutrient-efficient condition (Figure 5), which is usually characterized in the nutrient-deficient condition. As PCBP1 expression is shown to be broadly reduced in many types of tumor (Guo and Jia, 2018), our results here further demonstrate that PCBP1 represses intrinsic autophagic levels in both ovary tumors and colon tumors, indicating that p62 would be an general accompanying marker for other types of PCBP1-depleted tumors.

As an RNA-binding protein, PCBP1 can modulate multiple genes expressions on different expression levels, including RNA stability, RNA folding, splicing, translation, etc. Coordination regulation is also a conserved and efficient regulatory strategy to control expression of genes involved in sustaining organism homeostasis upon environmental changes. PCBP1, likely as a critical multifaceted gene may generally conduct such coordinated function via its RNA binding feature. Whether all these autophagy-related genes share a conserved RNA-binding sequence would be further investigated.

Currently, it is recognized that the exact role of autophagy in tumor progression is attributed to the component in tumor cells, tumor stages, physiological and tumor microenvironment (Levine, 2007; Mizushima, 2007; Galluzzi et al., 2015). At the early stages of tumorigenesis, when tumor vascular supply is limit, autophagy serves as a pro-survival mechanism for tumor cell by consuming its own unnecessary materials (Degenhardt et al., 2006; Levine, 2007; Huang et al., 2014). In line with this, our results thus have shown that for the tumor progression, in some circumstances, PCBP1 expression is inevitably repressed to induce relatively high autophagic level (Figures 1B, 7B).

Our results also demonstrate that there is no obvious PCBP1 expression difference among tumors from stage I + II groups to stage III + IV groups, but the clear deference between tumors and normal tissues (Figure 1F). Similarly, p62 expression is evidently reduced in tumors, compared with the normal tissues, but no clear alteration among tumors, regarding tumor stages and metastatic states (Figures 7B–E), indicating that PCBP1-p62 signaling axis may play repressive role only in the tumor initiation stage. Thus, PCBP1 would be an early tumor suppressor gene. However, the PCBP1 knock out animal model would be useful to validate whether a spontaneous tumorigenesis will occur along with aging. In addition, PCBP1 not only functions via autophagy, but other manners to interfere with tumor progression, for example, tumor cell cycle modulation (Shi et al., 2018), as tumor cell cycle can be modulated by particular autophagy during cancer development and by therapy (Zheng et al., 2019).

**FIGURE 8:**
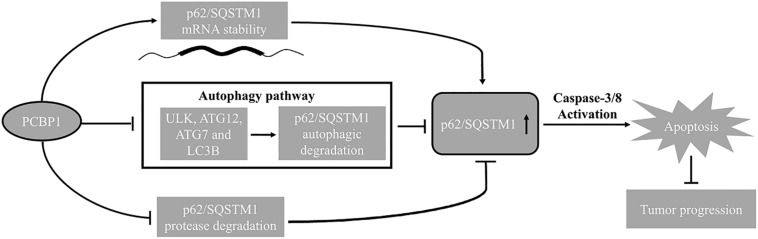
Schematic model of PCBP1 in regulating p62/SQSTM1 to repress tumor progression.

Interplay between autophagy and apoptosis is another complex phenomenon. Recent study disclosed that p62 plays a bridge role in autophagy and apoptosis through its adaptor protein feature (Moscat and Diaz-Meco, 2009). p62, as a substrate of autophagy, is usually used as a marker to examine autophagy completion status. Because p62 contains several interaction domains to many signaling molecules for their proteasomal degradation (Bardag-Gorce et al., 2005; Moscat and Diaz-Meco, 2009; Zheng et al., 2019), monitoring p62 degradation thus cannot accurately evaluate the exact autophagic flux or autophagy outcome. In line with this notion, our results here show more evidence that p62 can be regulated by three different ways by PCBP1, including inhibitions of both autophagy- and proteasome-mediated p62 degradation, as well as increase p62 mRNA stability (Figures 4, 8), which further indicates that p62 is not an ideal marker for detection of autophagy maturation. On the other hand, it is known that p62 can associate with caspase-8 and the subsequent apoptotic pathway (Jin et al., 2009; Yan et al., 2019). Likewise, our results here explain the reason why PCBP1 downregulation or depletion would be an important strategy to tumor cell survival by downregulations of p62 and caspase-8 (Figure 7 and Supplementary Figure 6). Whether this hypothesis is valid, future work needs to be further studied in the appropriate tumor models.

Overall, our results provide a potential concept that autophagy inhibition could be an efficient strategy to induce malignant tumors to undergo apoptotic cell death, when PCBP1 depletion. In line with our results, it is reported that inhibition of autophagy with both 3-MA and CQ leads to apoptosis in both ovarian and colorectal cancer cells (Huang et al., 2014; Lu et al., 2014; Tang et al., 2015).

In summary, combining our current and previous results, we conclude that PCBP1 plays a coordinate role in inhibiting tumorigenesis via blocking autophagy, and in turn promoting tumor cell apoptosis (Figure 8). Therefore, inhibition of the autophagy in PCBP1-depleted tumors could be a promising therapeutic strategy.

## Data Availability Statement

All datasets generated for this study are included in the article/Supplementary Material, further inquiries can be directed to the corresponding authors.

## Ethics Statement

The studies involving human participants were reviewed and approved by the Sun Yat-sen University Research Ethics Committee. The patients/participants provided their written informed consent to participate in this study.

## Author Contributions

HW, SY, and WZ conceived and designed the project. WZ, SZ, WG, and ZH conducted and collected the experiments and data interpretation. WZ, JK, and CH performed the statistical analysis. WZ, SZ, SY, and HW wrote and edited the manuscript. HW and SY supervised the project. All authors read and gave their approval for the final version of the manuscript.

## Conflict of Interest

The authors declare that the research was conducted in the absence of any commercial or financial relationships that could be construed as a potential conflict of interest.

## Supplementary Material

FIGURE S1 Scores of PCBP1, p62, and caspase 8 expression based on IHC staining intensity.

FIGURE S2 PCBP1 protein expression in the paired adjacent normal and tumor regions of human colorectal cancer. **(A)** Representative IHC staining of PCBP1 protein expression in the paired adjacent normal and tumor regions of colorectal. Scale bars are equal to 50 μm. **(B)** Statistical analysis of PCBP1 in 10 paired fresh normal and malignant tissues of colorectal tissues. The paired Student’s *t*-test was carried out to check the group difference. *p*-values are shown.

FIGURE S3 Endogenous PCBP1 knockdown increases autophagic flux. **(A)** Immunoblots of PCBP1 in the A2780, SKOV3, DLD-1, and HCT-116 cell lines. **(B)** Immunoblots of PCBP1 by the specific siRNA transfection. **(C)** Western blots of LC3 B in the indicated cells with GFP-PCBP1 (PCBP1) and the GFP-expressing control cells. GAPDH is used as a loading control. Protein bands’ intensity ratio of LC3II to GAPDH were quantified and normalized, and shown under each lane, respectively.

FIGURE S4 Genes precipitated in PCBP1 antibody-mediated RNA IP. The bound mRNAs are identified by RNA sequencing. Copy number of each gene is indicated in *Y*-axis, and the total number of PCBP1-bound mRNAs are shown in *X*-axis. –Autophagy-related ATG7 and ATG 12 are shown in the lower panel in red.

FIGURE S5 **(A,B)** CCK8 cell proliferation analyses of A2780 **(A)** and DLD-1 **(B)** cells with exogenous PCBP1 in the absence or presence of autophagic inhibitor CQ at 50 μM. Data presented are mean ± SD. NS, No Significance; **P* < 0.05; ***P* < 0.01; ****P* < 0.001, *n* = 3.

FIGURE S6 Relevance of PCBP1 to p62 and caspase 8 in ovary cancers. **(A)** Representative immunohistochemistry (IHC) analysis of p62 expression in the paired adjacent normal tissues and ovarian tumor samples. All images are photographed with 200X amplification. Scale bars in Supplementary Figures 6A,E equal to 100 μm. **(B)** Statistical comparison of p62 expression between primary and metastatic tumor tissues. **(C)** Representative immunohistochemistry (IHC) analysis of PCBP1, p62, and caspase 8 expressions in the same typical patient stages I–III. All Images are photographed with 200X amplification. Scale bars equal to 100 μm. **(D)** Correlationship of PCBP1 expression to p62. **(E)** Correlationship of PCBP1 expression to caspase-8. **(F)** Correlationship of p62 expression to caspase-8. **(G)** Correlationships among PCBP1, p62, and caspase 8 expressions in 90 ovary tumor samples.

TABLE S1 Primers used for RT-PCR amplification in this study.

## Abbreviations

3-MA: 3-Methyladenine
Act D: Actinomycin D
ANOVA: Analysis of Variance
A 5TG: Autophagy-related gene
CQ: Chloroquine diphosphate salts
IHC: I b mmunohistochemistry
KD: knock down
LC3B: Microtubule-associated protein Light Chain 3B
OCs: Ovarian cancer specimens
p62: p62/SQSTM1
PARP: Poly (ADP-ribose) polymerase
PCBP1: Poly C Binding Protein 1
PFA: Paraformaldehyde
RT-PCR: Reverse transcriptase-polymerase chain reaction
SDS-PAGE: SDS-polyacrylamide gel electrophoresis.

## References

[B1] Bardag-Gorce F. Francis T. Nan L. Li J. He L. Y. French B. A. (2005). Modifications in P62 occur due to proteasome inhibition in alcoholic liver disease. *Life Sci.* 77 2594–2602. 10.1016/j.lfs.2005.04.020 15964033

[B2] Bialik S. Dasari S. K. Kimchi A. (2018). Autophagy-dependent cell death - where, how and why a cell eats itself to death. *J. Cell Sci.* 131:152. 10.1242/jcs.215152 30237248

[B3] Bjorkoy G. Lamark T. Brech A. Outzen H. Perander M. Overvatn A. (2005). p62/SQSTM1 forms protein aggregates degraded by autophagy and has a protective effect on huntingtin-induced cell death. *J. Cell Biol.* 171 603–614. 10.1083/jcb.200507002 16286508 PMC2171557

[B4] D’Arcy M. S. (2019). Cell death: a review of the major forms of apoptosis, necrosis and autophagy. *Cell Biol. Int.* 43 582–592. 10.1002/cbin.11137 30958602

[B5] Degenhardt K. Mathew R. Beaudoin B. Bray K. Anderson D. Chen G. (2006). Autophagy promotes tumor cell survival and restricts necrosis, inflammation, and tumorigenesis. *Cancer Cell* 10 51–64. 10.1016/j.ccr.2006.06.001 16843265 PMC2857533

[B6] Fulda S. Debatin K. M. (2006). Extrinsic versus intrinsic apoptosis pathways in anticancer chemotherapy. *Oncogene* 25 4798–4811. 10.1038/sj.onc.1209608 16892092

[B7] Galluzzi L. Pietrocola F. Bravo-San P. J. Amaravadi R. K. Baehrecke E. H. Cecconi F. (2015). Autophagy in malignant transformation and cancer progression. *EMBO J.* 34 856–880. 10.15252/embj.201490784 25712477 PMC4388596

[B8] Giles K. M. Daly J. M. Beveridge D. J. Thomson A. M. Voon D. C. Furneaux H. M. (2003). The 3’-untranslated region of p21WAF1 mRNA is a composite cis-acting sequence bound by RNA-binding proteins from breast cancer cells, including HuR and poly(C)-binding protein. *J. Biol. Chem.* 278 2937–2946. 10.1074/jbc.M208439200 12431987

[B9] Guo J. Jia R. (2018). Splicing factor poly(rC)-binding protein 1 is a novel and distinctive tumor suppressor. *J. Cell. Physiol.* 234 33–41. 10.1002/jcp.26873 30132844

[B10] Guo J. Zhu C. Yang K. Li J. Du N. Zong M. (2017). Poly(C)-binding protein 1 mediates drug resistance in colorectal cancer. *Oncotarget* 8 13312–13319. 10.18632/oncotarget.14516 28076324 PMC5355098

[B11] Huang S. Okamoto K. Yu C. Sinicrope F. A. (2013). p62/sequestosome-1 up-regulation promotes ABT-263-induced caspase-8 aggregation/activation on the autophagosome. *J. Biol. Chem.* 288 33654–33666. 10.1074/jbc.M113.518134 24121507 PMC3837112

[B12] Huang Y. H. Al-Aidaroos A. Q. Yuen H. F. Zhang S. D. Shen H. M. Rozycka E. (2014). A role of autophagy in PTP4A3-driven cancer progression. *Autophagy* 10 1787–1800. 10.4161/auto.29989 25136802 PMC4198363

[B13] Ishii T. Hayakawa H. Igawa T. Sekiguchi T. Sekiguchi M. (2018). Specific binding of PCBP1 to heavily oxidized RNA to induce cell death. *Proc. Natl. Acad. Sci. U.S.A.* 115 6715–6720. 10.1073/pnas.1806912115 29891675 PMC6042155

[B14] Janku F. McConkey D. J. Hong D. S. Kurzrock R. (2011). Autophagy as a target for anticancer therapy. *Nat. Rev. Clin. Oncol.* 8 528–539. 10.1038/nrclinonc.2011.71 21587219

[B15] Ji F. J. Wu Y. Y. An Z. Liu X. S. Jiang J. N. Chen F. F. (2017). Expression of both poly r(C) binding protein 1 (PCBP1) and miRNA-3978 is suppressed in peritoneal gastric cancer metastasis. *Sci. Rep.* 7:15488 10.1038/s41598-017-15448-15449 PMC5686074 29138420

[B16] Jin Z. Li Y. Pitti R. Lawrence D. Pham V. C. Lill J. R. (2009). Cullin3-based polyubiquitination and p62-dependent aggregation of caspase-8 mediate extrinsic apoptosis signaling. *Cell* 137 721–735. 10.1016/j.cell.2009.03.015 19427028

[B17] Klionsky D. J. Abdalla F. C. Abeliovich H. Abraham R. T. Acevedo-Arozena A. Adeli K. (2012). Guidelines for the use and interpretation of assays for monitoring autophagy. *Autophagy* 8 445–544. 22966490 10.4161/auto.19496 PMC3404883

[B18] Kruidering M. Evan G. I. (2000). Caspase-8 in apoptosis: the beginning of “the end”? *Iubmb. Life* 50 85–90. 10.1080/713803693 11185963

[B19] Levine B. (2007). Cell biology: autophagy and cancer. *Nature* 446 745–747. 10.1038/446745a 17429391

[B20] Li J. Yuan J. (2008). Caspases in apoptosis and beyond. *Oncogene* 27 6194–6206. 10.1038/onc.2008.297 18931687

[B21] Lu Z. Baquero M. T. Yang H. Yang M. Reger A. S. Kim C. (2014). DIRAS3 regulates the autophagosome initiation complex in dormant ovarian cancer cells. *Autophagy* 10 1071–1092. 10.4161/auto.28577 24879154 PMC4091169

[B22] Maiuri M. C. Criollo A. Kroemer G. (2010). Crosstalk between apoptosis and autophagy within the Beclin 1 interactome. *EMBO J.* 29 515–516. 10.1038/emboj.2009.377 20125189 PMC2830702

[B23] Mizushima N. (2007). Autophagy: process and function. *Genes Dev.* 21 2861–2873. 10.1101/gad.1599207 18006683

[B24] Moscat J. Diaz-Meco M. T. (2009). p62 at the crossroads of autophagy, apoptosis, and cancer. *Cell* 137 1001–1004. 10.1016/j.cell.2009.05.023 19524504 PMC3971861

[B25] Nishinakamura H. Minoda Y. Saeki K. Koga K. Takaesu G. Onodera M. (2007). An RNA-binding protein alphaCP-1 is involved in the STAT3-mediated suppression of NF-kappaB transcriptional activity. *Int. Immunol.* 19 609–619. 10.1093/intimm/dxm026 17383969

[B26] Pankiv S. Clausen T. H. Lamark T. Brech A. Bruun J. A. Outzen H. (2007). p62/SQSTM1 binds directly to Atg8/LC3 to facilitate degradation of ubiquitinated protein aggregates by autophagy. *J. Biol. Chem.* 282 24131–24145. 10.1074/jbc.M702824200 17580304

[B27] Ravikumar B. Sarkar S. Davies J. E. Futter M. Garcia-Arencibia M. Green-Thompson Z. W. (2010). Regulation of mammalian autophagy in physiology and pathophysiology. *Physiol. Rev.* 90 1383–1435. 10.1152/physrev.00030.2009 20959619

[B28] Shi H. Li H. Yuan R. Guan W. Zhang X. Zhang S. (2018). PCBP1 depletion promotes tumorigenesis through attenuation of p27(Kip1) mRNA stability and translation. *J. Exp. Clin. Cancer Res.* 37:187 10.1186/s13046-018-0840-841 PMC6081911 30086790

[B29] Singh S. S. Vats S. Chia A. Y. Tan T. Z. Deng S. Ong M. S. (2018). Dual role of autophagy in hallmarks of cancer. *Oncogene* 37 1142–1158. 10.1038/s41388-017-0046-46 29255248

[B30] Tang Y. Li M. Wang Y. L. Threadgill M. D. Xiao M. Mou C. F. (2015). ART1 promotes starvation-induced autophagy: a possible protective role in the development of colon carcinoma. *Am. J. Cancer Res.* 5 498–513. 25973293 PMC4396040

[B31] Tao M. Liu T. You Q. Jiang Z. (2020). p62 as a therapeutic target for tumor. *Eur. J. Med. Chem.* 193:112231. 10.1016/j.ejmech.2020.112231 32193054

[B32] Tripathi V. Sixt K. M. Gao S. Xu X. Huang J. Weigert R. (2016). Direct Regulation of Alternative Splicing by SMAD3 through PCBP1 Is Essential to the Tumor-Promoting Role of TGF-beta. *Mol. Cell.* 64 549–564. 10.1016/j.molcel.2016.09.013 27746021 PMC5123764

[B33] Valente G. Morani F. Nicotra G. Fusco N. Peracchio C. Titone R. (2014). Expression and clinical significance of the autophagy proteins BECLIN 1 and LC3 in ovarian cancer. *Biomed. Res. Int.* 2014:462658. 10.1155/2014/462658 25136588 PMC4127242

[B34] Villa E. Proics E. Rubio-Patino C. Obba S. Zunino B. Bossowski J. P. (2017). Parkin-independent mitophagy controls chemotherapeutic response in cancer cells. *Cell Rep.* 20 2846–2859. 10.1016/j.celrep.2017.08.087 28930681

[B35] Wang H. Vardy L. A. Tan C. P. Loo J. M. Guo K. Li J. (2010). PCBP1 suppresses the translation of metastasis-associated PRL-3 phosphatase. *Cancer Cell* 18 52–62. 10.1016/j.ccr.2010.04.028 20609352

[B36] Wang X. Guo J. Che X. Jia R. (2019). PCBP1 inhibits the expression of oncogenic STAT3 isoform by targeting alternative splicing of STAT3 exon 23. *Int. J. Biol. Sci.* 15 1177–1186. 10.7150/ijbs.33103 31223278 PMC6567812

[B37] Wang Z. Yin W. Zhu L. Li J. Yao Y. Chen F. (2018). Iron drives T helper cell pathogenicity by promoting RNA-binding protein PCBP1-mediated proinflammatory cytokine production. *Immunity* 49 80–92. 10.1016/j.immuni.2018.05.008 29958803

[B38] Wu H. Che X. Zheng Q. Wu A. Pan K. Shao A. (2014). Caspases: a molecular switch node in the crosstalk between autophagy and apoptosis. *Int. J. Biol. Sci.* 10 1072–1083. 10.7150/ijbs.9719 25285039 PMC4183927

[B39] Yan X. Y. Zhong X. R. Yu S. H. Zhang L. C. Liu Y. N. Zhang Y. (2019). p62 aggregates mediated Caspase 8 activation is responsible for progression of ovarian cancer. *J. Cell Mol. Med.* 23 4030–4042. 10.1111/jcmm.14288 30941888 PMC6533521

[B40] Young M. M. Takahashi Y. Khan O. Park S. Hori T. Yun J. (2012). Autophagosomal membrane serves as platform for intracellular death-inducing signaling complex (iDISC)-mediated caspase-8 activation and apoptosis. *J. Biol. Chem.* 287 12455–12468. 10.1074/jbc.M111.309104 22362782 PMC3320995

[B41] Zhang M. P. Zhang W. S. Tan J. Zhao M. H. Lian L. J. Cai J. (2017a). Poly r(C) binding protein (PCBP) 1 expression is regulated at the post-translation level in thyroid carcinoma. *Am. J. Transl. Res.* 9 708–714. 28337299 PMC5340706

[B42] Zhang M. P. Zhang W. S. Tan J. Zhao M. H. Lian L. J. Cai J. (2017b). Poly r(C) binding protein (PCBP) 1 expression is regulated by the E3 ligase UBE4A in thyroid carcinoma. *Biosci. Rep.* 37:114. 10.1042/BSR20170114 28963376 PMC5662924

[B43] Zhang T. Huang X. H. Dong L. Hu D. Ge C. Zhan Y. Q. (2010). PCBP-1 regulates alternative splicing of the CD44 gene and inhibits invasion in human hepatoma cell line HepG2 cells. *Mol. Cancer* 9:72. 10.1186/1476-4598-9-72 20361869 PMC2864215

[B44] Zhang W. Shi H. Zhang M. Liu B. Mao S. Li L. (2016). Poly C binding protein 1 represses autophagy through downregulation of LC3B to promote tumor cell apoptosis in starvation. *Int. J. Biochem. Cell Biol.* 73 127–136. 10.1016/j.biocel.2016.02.009 26880484

[B45] Zhao L. Wang Z. G. Zhang P. Yu X. F. Su X. J. (2019). Poly r(C) binding protein 1 regulates posttranscriptional expression of the ubiquitin ligase TRIM56 in ovarian cancer. *IUBMB Life* 71 177–182. 10.1002/iub.1948 30281912

[B46] Zhao Y. Chen S. Gou W. F. Xiao L. J. Takano Y. Zheng H. C. (2014). Aberrant Beclin 1 expression is closely linked to carcinogenesis, differentiation, progression, and prognosis of ovarian epithelial carcinoma. *Tumour Biol.* 35 1955–1964. 10.1007/s13277-013-1261-1266 24132590

[B47] Zheng K. He Z. Kitazato K. Wang Y. (2019). Selective autophagy regulates cell cycle in cancer therapy. *Theranostics* 9 104–125. 10.7150/thno.30308 30662557 PMC6332805

