# Supplementary material for: Poly C Binding Protein 1 Regulates p62/SQSTM1 mRNA Stability and Autophagic Degradation to Repress Tumor Progression
Source: Front Genet. 2020 Aug 14;11:930. doi: 10.3389/fgene.2020.00930 (PMC7457068; doi:10.3389/fgene.2020.00930)
Supplement: FIGURE S1 — Scores of PCBP1, p62, and caspase 8 expression based on IHC staining intensity. [file Data_Sheet_1.PDF]

Supplementary Table 1. Primers used for RT-PCR amplification in this study.

Primer Name	Sequence
GADPH-Forward	5’-ACCACAGTCCATGCCATCA -3’
GADPH-Reverse	5’-TCCACCACCCTGTTGCTGTA -3’
ATG7-Forward	5’-GATGGAGAGCTCCTCAGCA -3’
ATG7-Reverse	5’-ATTGCTGCATCAAGAAACCC -3’
ULK1-Forward	5’-AAGAGCCTGATGGTGTCCTC -3’
ULK1 -Reverse	5’-GTGGCCCTGTACGACTTCC -3’
PRL3-Forward	5’-AGAAGTACGGGGCTACCACTG -3’
PRL3 -Reverse	5’-CGCTCTCAATAAGCGCCAG -3’
ATG12-Forward	5’-TTGTGGCCTCAGAACAGTTG -3’
ATG12 -Reverse	5’-CCATCACTGCCAAAACACTC -3’
p62-Forward	5’-TACAAGGGAAGTGGCTATC-3’
p62 -Reverse	5’-TTACACTGACAATTTCATCC-3’

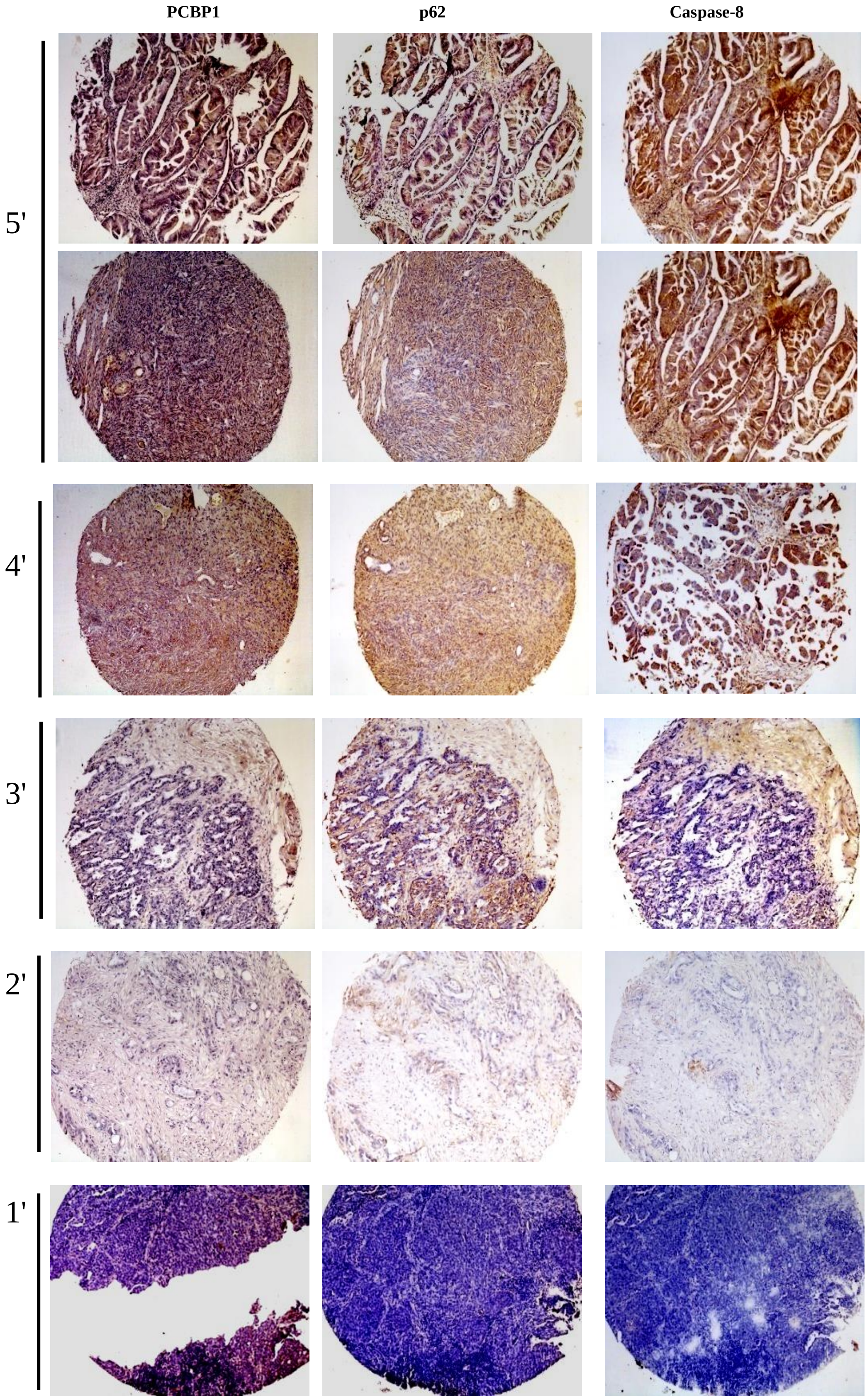

Supplementary Figure 1. Scores of PCBP1, p62 and caspase 8 expression based on IHC staining intensity.

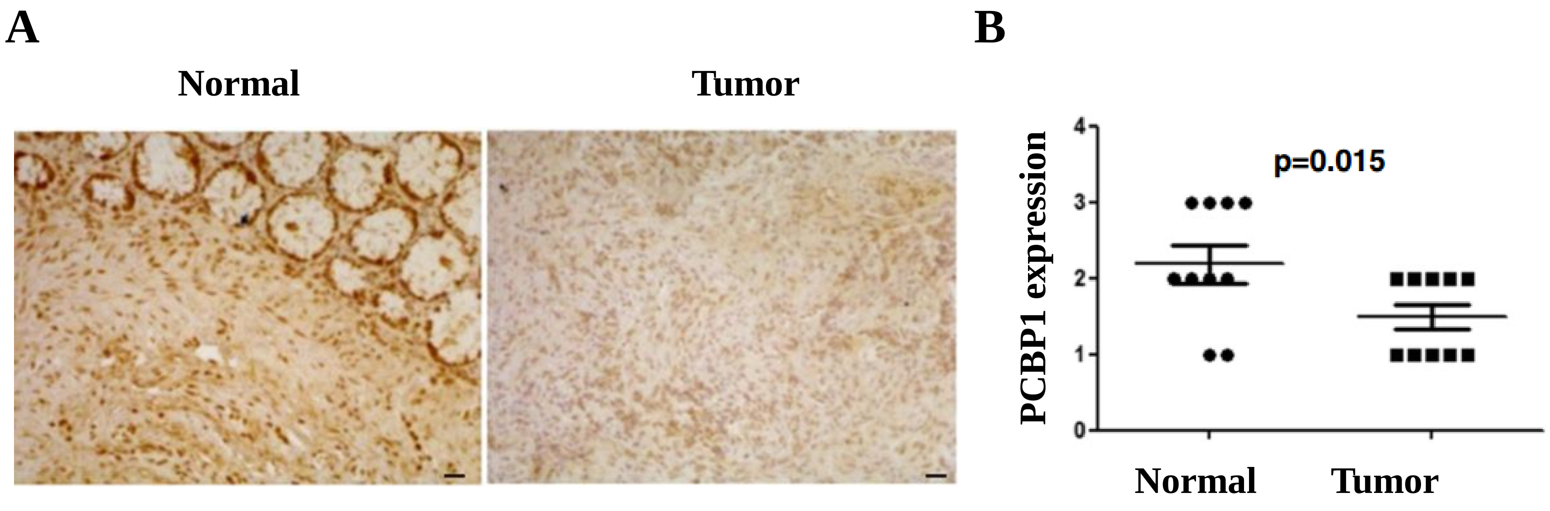

Supplementary Figure 2. PCBP1 protein expression in the paired adjacent normal and tumor regions of human colorectal cancer. (A) Representative IHC staining of PCBP1 protein expression in the paired adjacent normal and tumor regions of colorectal. Scale bars are equal to 50 μm. (B) Statistical analysis of PCBP1 in 10 paired fresh normal and malignant tissues of colorectal tissues. The paired student t test was carried out to check the group difference. p values are shown.

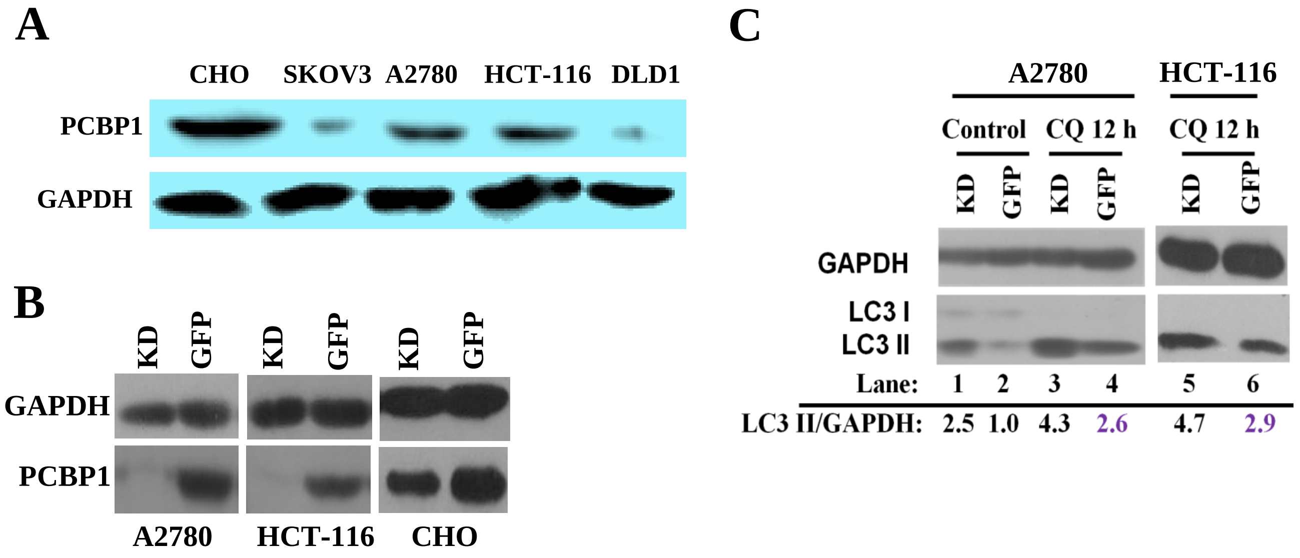

Supplementary Figure 3. Endogenous PCBP1 knockdown increases autophagic flux.

(A) Immunoblosts of PCBP1 in the A2780, SKOV3, DLD-1 and HCT-116 cell lines.

(B) Immunoblots of PCBP1 by the specific siRNA transfection;

(C) Western blots of LC3 B in the indicated cells with GFP-PCBP1 (PCBP1) and the GFP-expressing control cells. GAPDH is used as a loading control. Protein bands' intensity ratio of LC3II to GAPDH were quantified and normalized, and shown under each lane, respectively.

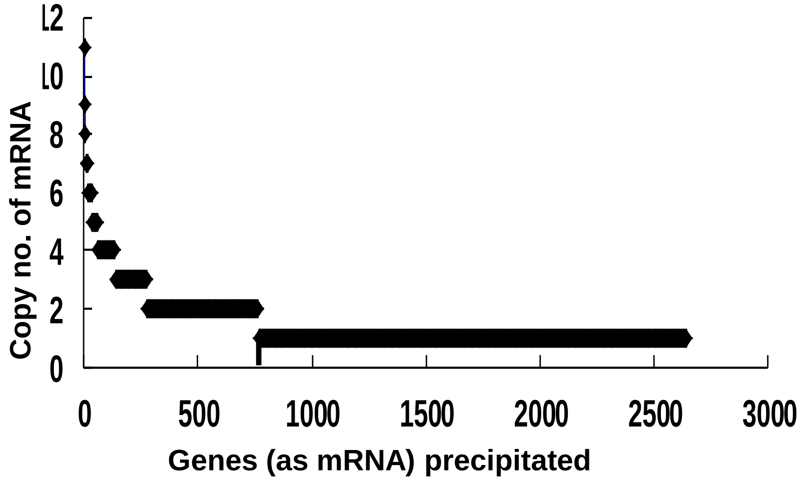

300	Homo sapiens ataxin 10, mRNA (cDNA clone MGC:4152 IMAGE:3030062), complete cds	2
301	Homo sapiens ATG12 autophagy related 12 homolog (S. cerevisiae), mRNA (cDNA clone IMAGE:4103628), complete cds	2
302	Homo sapiens ATP synthase 6 mRNA, complete cds; mitochondrial gene for mitochondrial product	2
891	Homo sapiens ataxin 2, mRNA (cDNA clone IMAGE:40027338), partial cds	1
892	Homo sapiens ATG7 autophagy related 7 homolog (S. cerevisiae), mRNA (cDNA clone MGC:1334 IMAGE:3504204), complete cds	1

Supplementary Figure 4. Genes precipitated in PCBP1 antibody-mediated RNA IP. The bound mRNAs are identified by RNA sequencing. Copy number of each gene is indicated in Y-axis, and the total number of PCBP1-bound mRNAs are shown in X-axis. – Autophagy-related ATG7 and ATG 12 are shown in the lower panel in red.

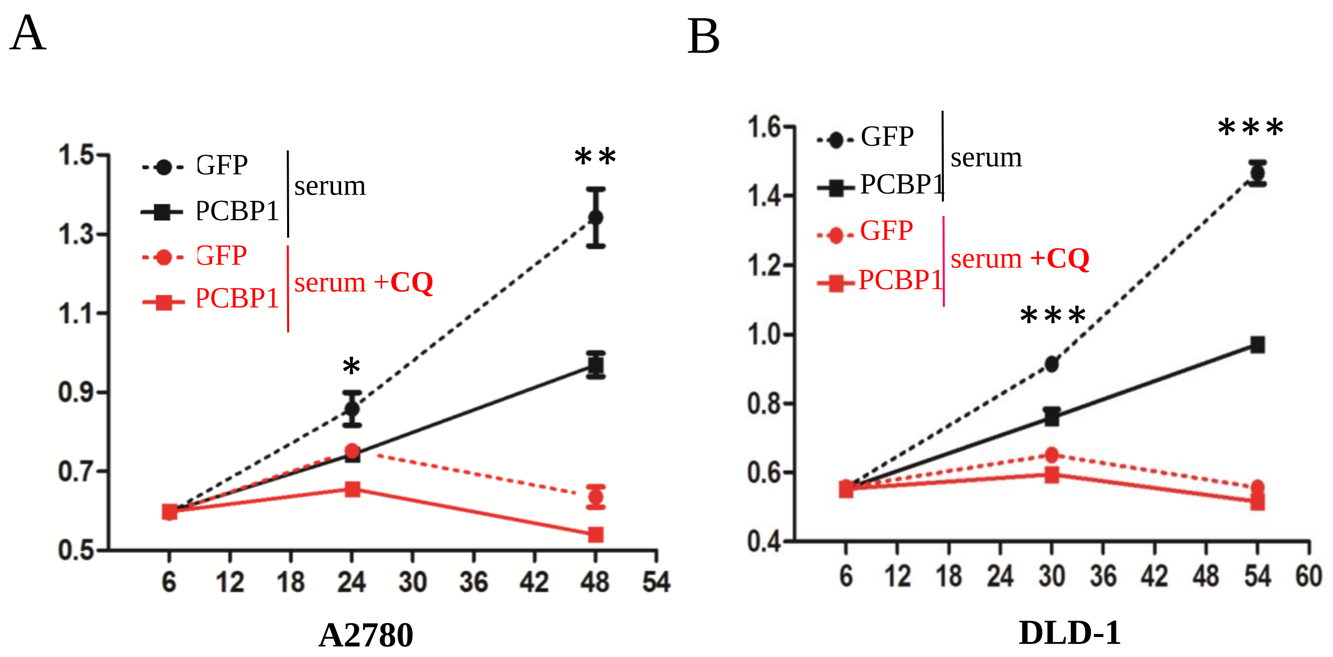

Supplementary Figure 5. (A) and (B) CCK8 cell proliferation analyses of A2780 (**A**) and DLD-1 (**B**) cells with exogenous PCBP1 in the absence or presence of autophagic inhibitor CQ at 50 μM. Data presented are mean ±SD. NS: No Significance; * P < 0.05; ** P < 0.01; *** P < 0.001, n=3.

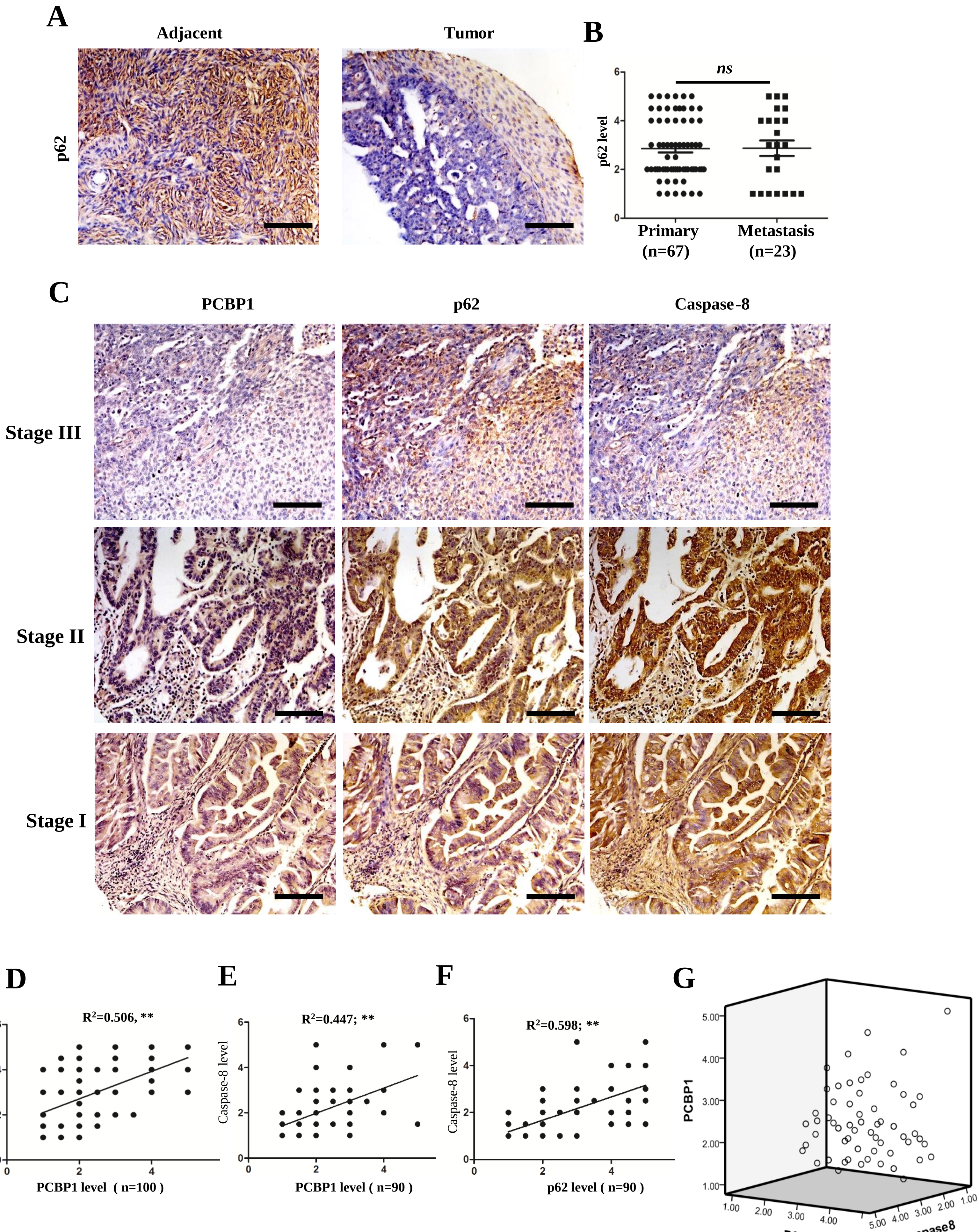

Supplementary Figure 6

Supplementary Figure 6. Relevance of PCBP1 to p62 and caspase 8 in ovary cancers.

(A) Representative immunohistochemistry (IHC) analysis of p62 expression in the paired adjacent normal tissues and ovarian tumor samples. All pictures are photographed with 200X amplification. Scale bars are equal to 100 μm.

(B) The statistical comparison of p62 expression between primary and metastatic tumor tissues .

(C) Representative immunohistochemistry (IHC) analysis of PCBP1, p62 and caspase 8 expressions in the same typical patient stage I-III. All pictures are photographed with 200X amplification. Scale bars are equal to 100 μm.

(D) Correlationship of PCBP1 expression to p62.

(E) Correlationship of PCBP1 expression to caspase-8.

(F) Correlationship of p62 expression to caspase-8.

(G) Schematic correlationships among PCBP1, p62 and caspase 8 expressions in 90 ovary tumor samples.
